# Supplementary figures and images for: Acute vaping exacerbates microbial pneumonia due to calcium (Ca2+) dysregulation
Source: PLoS One. 2021 Aug 12;16(8):e0256166. doi: 10.1371/journal.pone.0256166 (PMC8360547; doi:10.1371/journal.pone.0256166)

## Slide 1
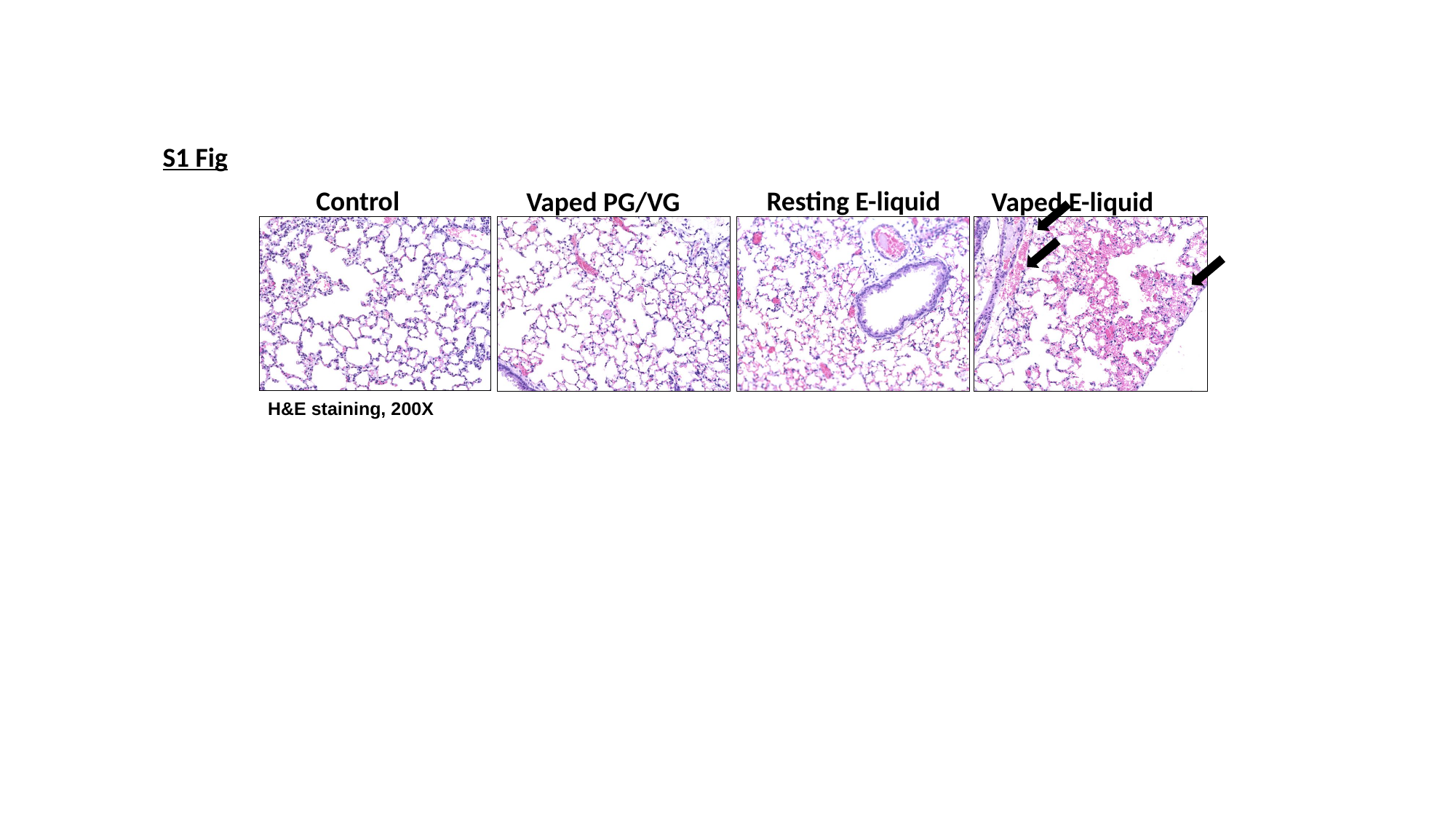

S1 Fig
Control
Resting E-liquid
Vaped PG/VG
Vaped E-liquid
H&E staining, 200X

Supplement: S1 Fig — Mice received either PBS, vaped PG/VG vehicle, resting e-liquid or vaped e-liquid (10 μl) once daily intranasal (IN) for 3 days and were then euthanized. n = 4 mice per treatment group. H&E staining (200X magnification) of sections of lung tissue isolated from mock-, vaped vehicle-, resting e-liquid- or vaped e-liquid- treated mice. Alveolar wall thickening was particularly observable in the lungs from the vaped e-liquid-treated mice compared to the mock control. Arrows indicate alveoli thickening and neutrophil influx. (PPTX) [file pone.0256166.s001.pptx]

## Slide 1
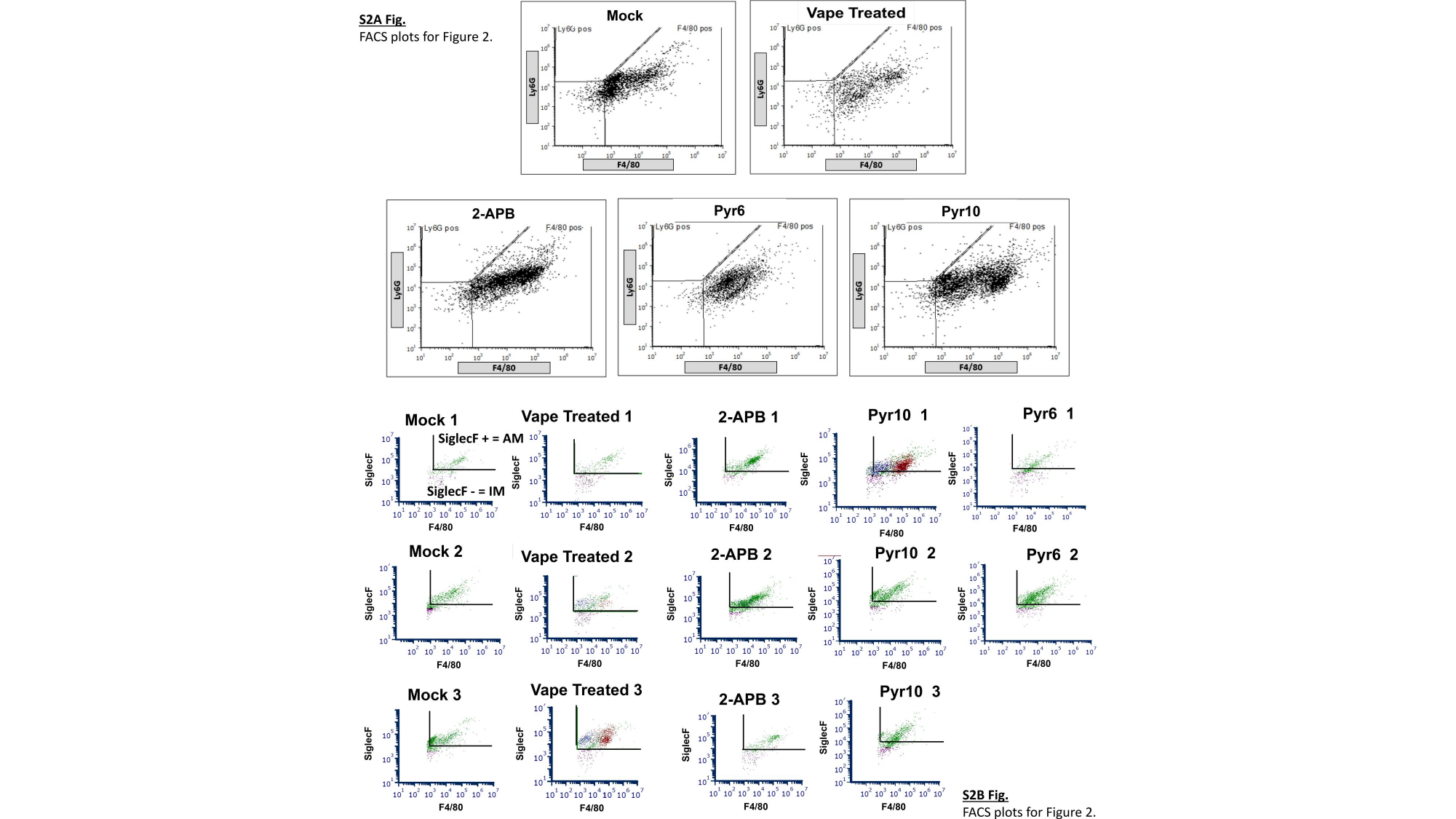

Supplement: S2 Fig — (A) Accompanying FACS plot data for Fig 2. (B) Multiple FACS scatter plot data frames to accompany Fig 2C. AM = SiglecF+/F4/80+ and IM = SiglecF-/F4/80+. AM = alveolar macrophage, IM = interstitial macrophage. (PPTX) [file pone.0256166.s002.pptx]

## Slide 1
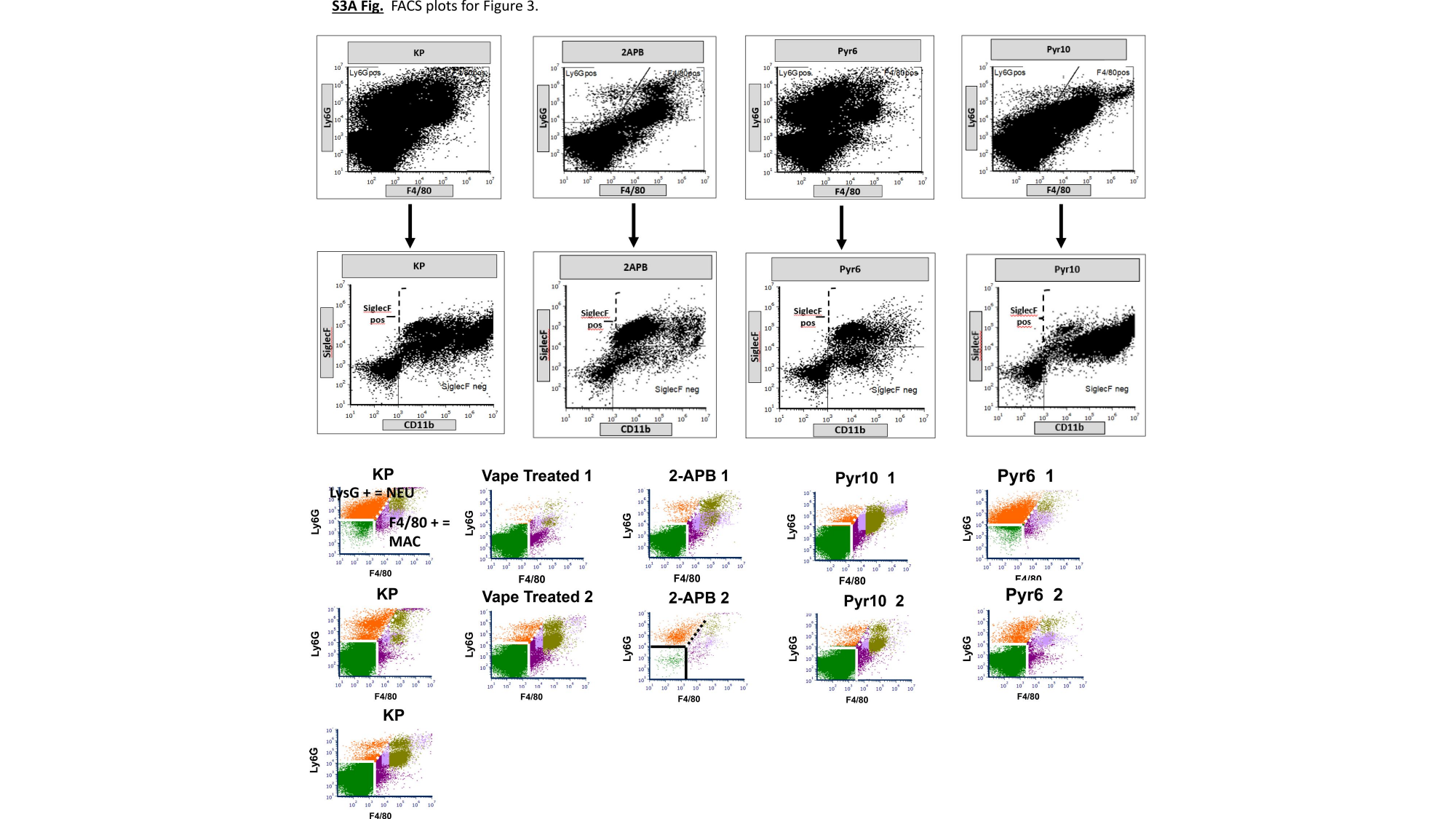

Supplement: S3 Fig — (A) Accompanying FACS plot data for Fig 3. Includes data for all drug treatment groups (2-APB, Pyr-6 and Pyr-10) and K. pneumoniae only. (B) Multiple FACS scatter plot data frames to accompany Fig 3C. MAC = F4/80+ and NEU = LysG+. MAC = macrophage, NEU = neutrophil. (PPTX) [file pone.0256166.s003.pptx]
