# Supplementary material for: Acute vaping exacerbates microbial pneumonia due to calcium (Ca2+) dysregulation
Source: PLoS One. 2021 Aug 12;16(8):e0256166. doi: 10.1371/journal.pone.0256166 (PMC8360547; doi:10.1371/journal.pone.0256166)
Supplement: Attachment — Submitted filename: Response to Reviewers.docx [file pone.0256166.s004.docx]

PONE-D-21-13261

Acute Vaping Exacerbates Microbial Pneumonia due to Calcium (Ca2+) Dysregulation

PLOS ONE

First of all, we thank the Editor and the reviewers for their comments and guidance, which have allowed us to revise and improve our manuscript. We have now addressed each and every issue raised by both of the reviewers. Our comments to the reviewers are below in **green** text, and we include a marked-up copy of our manuscript that highlights changes made to the original version as requested. The **(1)** “Marked Up” version of the manuscript has changes highlighted in both red and blue. Line numbers refer to lines within the “Marked Up” doc. We also include an **(2)** unmarked version that does not include “Track changes”.

Also, please update our funding statement to read: "This work was supported in part by a grant from the National Cancer Institute (NCI) NIH 5-U54-CA156733-10, National Minority Health Disparities Institute (NIMHD) NIH-3-U54MD012392 and by funds from NCCU BRITE."

Thank you,

Dear Dr. Sivaraman,

Thank you for submitting your manuscript to PLOS ONE. After careful consideration, we feel that it has merit but does not fully meet PLOS ONE’s publication criteria as it currently stands. Therefore, we invite you to submit a revised version of the manuscript that addresses the points raised during the review process.

Please submit your revised manuscript by Aug 07 2021 11:59PM. If you will need more time than this to complete your revisions, please reply to this message or contact the journal office at plosone@plos.org. When you're ready to submit your revision, log on to https://www.editorialmanager.com/pone/ and select the 'Submissions Needing Revision' folder to locate your manuscript file.

Please include the following items when submitting your revised manuscript:

A rebuttal letter that responds to each point raised by the academic editor and reviewer(s). You should upload this letter as a separate file labeled 'Response to Reviewers'.

A marked-up copy of your manuscript that highlights changes made to the original version. You should upload this as a separate file labeled 'Revised Manuscript with Track Changes'.

An unmarked version of your revised paper without tracked changes. You should upload this as a separate file labeled 'Manuscript'.

If you would like to make changes to your financial disclosure, please include your updated statement in your cover letter. Guidelines for resubmitting your figure files are available below the reviewer comments at the end of this letter.

If applicable, we recommend that you deposit your laboratory protocols in protocols.io to enhance the reproducibility of your results. Protocols.io assigns your protocol its own identifier (DOI) so that it can be cited independently in the future. For instructions see: http://journals.plos.org/plosone/s/submission-guidelines#loc-laboratory-protocols. Additionally, PLOS ONE offers an option for publishing peer-reviewed Lab Protocol articles, which describe protocols hosted on protocols.io. Read more information on sharing protocols at https://plos.org/protocols?utm_medium=editorial-email&utm_source=authorletters&utm_campaign=protocols.

We look forward to receiving your revised manuscript.

Kind regards,

Shama Ahmad, Ph.D.

Academic Editor

PLOS ONE

Journal Requirements:

When submitting your revision, we need you to address these additional requirements.

1. Please ensure that your manuscript meets PLOS ONE's style requirements, including those for file naming. The PLOS ONE style templates can be found at

https://journals.plos.org/plosone/s/file?id=wjVg/PLOSOne_formatting_sample_main_body.pdf and

https://journals.plos.org/plosone/s/file?id=ba62/PLOSOne_formatting_sample_title_authors_affiliations.pdf

2. We ask that you please remove citations for unavailable and unpublished work, including manuscripts that have been submitted but not yet accepted (e.g., “unpublished work,” “data not shown”). Instead, include those data as supplementary material or deposit the data in a publicly available database.

**Done. We have removed all “data not shown” and replaced with a citation for our recent publication. Lines 209 and 323.**

3. Thank you for your ethics statement: "This study was performed under authority of the NCCU IACUC. The approved protocol is:

VS-10-09-2019.

For studies, isofluorane was used for anesthesia, and inhaled CO2 was used to euthanasia, as per protocol."

**Please amend your current ethics statement to confirm that your named ethics committee specifically approved this study.**

**For additional information about PLOS ONE submissions requirements for ethics oversight of animal work, please refer to http://journals.plos.org/plosone/s/submission-guidelines#loc-animal-research**

**Once you have amended this/these statement(s) in the Methods section of the manuscript, please add the same text to the “Ethics Statement” field of the submission form (via “Edit Submission”).**

4. Thank you for stating the following in the Acknowledgments Section of your manuscript:

"This work was supported in part by a grant from the National Cancer Institute (NCI) NIH 5-U54-

CA156733-10, National Minority Health Disparities Institute (NIMHD) NIH-3-U54MD012392 and

by funds from NCCU BRITE."

We note that you have provided funding information that is not currently declared in your Funding Statement. However, funding information should not appear in the Acknowledgments section or other areas of your manuscript. We will only publish funding information present in the Funding Statement section of the online submission form.

Please remove any funding-related text from the manuscript and let us know how you would like to update your Funding Statement. Currently, your Funding Statement reads as follows:

"The author(s) received no specific funding for this work."

**Done. Please include the following text for our funding statement:**

**“This work was supported in part by a grant from the National Cancer Institute (NCI) NIH 5-U54-CA156733-10, National Minority Health Disparities Institute (NIMHD) NIH-3-U54MD012392 and by funds from NCCU BRITE.”**

Please include your amended statements within your cover letter; we will change the online submission form on your behalf.

5. We note that you have included the phrase “data not shown” in your manuscript. Unfortunately, this does not meet our data sharing requirements. PLOS does not permit references to inaccessible data. We require that authors provide all relevant data within the paper, Supporting Information files, or in an acceptable, public repository. Please add a citation to support this phrase or upload the data that corresponds with these findings to a stable repository (such as Figshare or Dryad) and provide and URLs, DOIs, or accession numbers that may be used to access these data. Or, if the data are not a core part of the research being presented in your study, we ask that you remove the phrase that refers to these data.

**We have removed all “data not shown” and replaced with a citation for our recent publication. Lines 209 and 323.**

[Note: HTML markup is below. Please do not edit.]

Reviewers' comments:

Reviewer's Responses to Questions

Comments to the Author

1. Is the manuscript technically sound, and do the data support the conclusions?

The manuscript must describe a technically sound piece of scientific research with data that supports the conclusions. Experiments must have been conducted rigorously, with appropriate controls, replication, and sample sizes. The conclusions must be drawn appropriately based on the data presented.

Reviewer #1: Yes

Reviewer #2: Partly

2. Has the statistical analysis been performed appropriately and rigorously?

Reviewer #1: N/A

Reviewer #2: Yes

3. Have the authors made all data underlying the findings in their manuscript fully available?

The PLOS Data policy requires authors to make all data underlying the findings described in their manuscript fully available without restriction, with rare exception (please refer to the Data Availability Statement in the manuscript PDF file). The data should be provided as part of the manuscript or its supporting information, or deposited to a public repository. For example, in addition to summary statistics, the data points behind means, medians and variance measures should be available. If there are restrictions on publicly sharing data—e.g. participant privacy or use of data from a third party—those must be specified.

Reviewer #1: Yes

Reviewer #2: Yes

4. Is the manuscript presented in an intelligible fashion and written in standard English?

PLOS ONE does not copyedit accepted manuscripts, so the language in submitted articles must be clear, correct, and unambiguous. Any typographical or grammatical errors should be corrected at revision, so please note any specific errors here.

Reviewer #1: Yes

Reviewer #2: Yes

5. Review Comments to the Author

Please use the space provided to explain your answers to the questions above. You may also include additional comments for the author, including concerns about dual publication, research ethics, or publication ethics. (Please upload your review as an attachment if it exceeds 20,000 characters)

Reviewer #1: This is a very well-written and surprisingly useful mouse study. The information is current, useful and important for interested pulmonary health providers. I have just a few thoughts.

Traditionally the materials and methods are placed before the results, correct? **Now corrected.**

Vaping resulting in acute cellular lung damage which increases the pathology process in mice infected with K.pneumoniae is important basic information. But why K.pneumoniae? Is it the mouse equivalent of pneumococcal in humans? **Excellent point by the reviewer. We now include text for our rationale of using *K. pneumoniae*. Lines 215-217.**

Reviewer #2: Zhang et al developed a mouse model of vaped e-liquid pulmonary exposure and studied the effects of calcium ion channel inhibitors in cohorts of mice that received either vaping or vaping+Klebsiella pneumonia. While some of the results are encouraging, the authors overstated their findings – the tested inhibitors barely showed therapeutic effects in vaping+bacterial pneumonia model.

**We have now amended the text to indicate the obtained data are promising but not complete and that further study will be required to understand this mechanism (Line 329-335).**

Please proof-read and correct typographical/grammar issues.

Abstract:

The authors wrote – “Using this model, we demonstrate through the use of clinical measures, that is, the lung wet:dry ratio and lung histology/H&E staining, lung pathology.” Although these parameters are clinically used but only in EVALI-related dead patients or post-mortem. These parameters are widely used in pre-clinical models. Lung histology and lung pathology can be synonymously used, therefore one of these two words can be deleted. **Edited and now done. Lines 50-52.**

The authors wrote – “This increase in macrophage activation appears to exacerbate pneumonia when due to pulmonary microbial infection.” Not clear, please paraphrase. **Edited. Lines 54-56.**

Results:

The authors wrote “To that end, we developed a protocol utilizing commercially available E-cig devices to aerosolize the e-liquid and then condensed the vapors back into a “vaped” e-liquid. With this new reagent in hand, we first assessed its toxicity in vitro (data not shown, manuscript under review).” In my opinion, the manuscript under review should be removed. **Removed and replaced. We now reference our recent publication (Sivaraman et al.,** ***Front. Physiol.,* 2021) for these data, which are now freely available. Lines 208-209.**

Page 6 – “lung pathology of the K. pneumoniae-infected only animals also demonstrated a mild inflammatory influx of neutrophils within the alveoli, though the majority of airways were unimpeded (Fig. 1B).” It should be Fig. 1C. **Done. Line 228.**

In figure 1B, on the y-axis, lung weight (mg) is written. Ratios have no units. **Done.**

In figure 1C, instead of pixilation, please use scoring methods such as ATS scoring for ALI An official American Thoracic Society workshop report: features and measurements of experimental acute lung injury in animals - PubMed (nih.gov) (PMID: 21531958). **ATS scoring for ALI has now been performed and is now included within Figure 1C. Please see also lines 187-193 and 243-244.**

In Figures 2A and 2B, please use consistent labeling for axes. Present vape+control bar after sham (control) group for easy interpretation here and subsequent bar graphs. **Done.**

If possible, please present bar graphs with scatter plots to see the variation of the data. **This is an excellent point. These data sets are, however, very large and were collected and analyzed by the NC Central University Flow Core. We did ask for and receive additional raw data scatter plots from the Core and now include these plots to allow for readers to observe the variation of the data (Supplementary Figures 2B and 3B). The quantitative data are currently within Figures 2 and 3.**

For ease of interpretation, please present the study paradigm (as in Figure 1A) in a panel for figures 2 and 3. By looking at legends for figures 2 and 3, it is difficult to differentiate the study paradigm. **Study paradigms are now included for Figures 2 and 3, i.e., Figures 2A and 3A.**

6. PLOS authors have the option to publish the peer review history of their article (what does this mean?). If published, this will include your full peer review and any attached files.

If you choose “no”, your identity will remain anonymous but your review may still be made public.

Do you want your identity to be public for this peer review? For information about this choice, including consent withdrawal, please see our Privacy Policy.

Reviewer #1: No

Reviewer #2: No

[NOTE: If reviewer comments were submitted as an attachment file, they will be attached to this email and accessible via the submission site. Please log into your account, locate the manuscript record, and check for the action link "View Attachments". If this link does not appear, there are no attachment files.]

While revising your submission, please upload your figure files to the Preflight Analysis and Conversion Engine (PACE) digital diagnostic tool, https://pacev2.apexcovantage.com/. PACE helps ensure that figures meet PLOS requirements. To use PACE, you must first register as a user. Registration is free. Then, login and navigate to the UPLOAD tab, where you will find detailed instructions on how to use the tool. If you encounter any issues or have any questions when using PACE, please email PLOS at figures@plos.org. Please note that Supporting Information files do not need this step.
